# Vigorous-intensity exercise as a modulator of cardiac adipose tissue in women with obesity: a cross-sectional and randomized pilot study

**DOI:** 10.3389/fendo.2023.1104441

**Published:** 2023-05-08

**Authors:** Sumsen Thapa, Bharath S. Selvaraj, Paige N. Davis, Bryan Smith, Amy H. Givan, Jose A. Perez-Rivera, Pamela K. Woodard, Jon D. Klingensmith, Maria Fernandez-del-Valle

**Affiliations:** ^1^ Department of Applied Health, Southern Illinois University Edwardsville, Edwardsville, IL, United States; ^2^ Iowa Digestive Disease Center, Heartland Medical Research, Inc., Clive, IA, United States; ^3^ Department of Cardiopulmonary Rehabilitation, Charleston Area Medical Center (CAMC) Memorial Hospital at West Virginia, Charleston, WV, United States; ^4^ Division of Rehabilitation Sciences, University of Texas Medical Branch at Galveston, Galveston, TX, United States; ^5^ Department of Cardiology, University Hospital of Burgos, Burgos, Spain; ^6^ Facultad de Ciencias de la Salud, Universidad Isabel I, Isabel, Spain; ^7^ Mallinckrodt Institute of Radiology, Washington University School of Medicine, St. Louis, MO, United States; ^8^ Department of Electrical and Computer Engineering, Southern Illinois University Edwardsville, Edwardsville, IL, United States; ^9^ Department of Functional Biology, University of Oviedo, Oviedo, Spain; ^10^ Health Research Institute of the Principality of Asturias (ISPA), Oviedo, Spain

**Keywords:** cardiac adipose tissue, obesity, physical fitness, vigorous physical activity, women

## Abstract

Cardiac adipose tissue (CAT) has become an important target for the reduction of disease risk. Supervised exercise programs have shown potential to "significantly" reduce CAT; however, the impact of different exercise modalities is not clear, and the relationships between CAT, physical activity (PA) levels and fitness (PFit) remain unknown. Therefore, the purpose of this study was to analyze the relationships between CAT, PA and PFit, and to explore the effects of different exercise modalities in a group of women with obesity. A total of 26 women (age: 23.41 ± 5.78 years-old) were enrolled in the cross-sectional study. PA, cardiorespiratory fitness, muscular strength, body composition and CAT were evaluated. The pilot intervention included 16 women randomized to a control (CON, n=5), high intensity interval training (HIIT, n = 5) and high-intensity circuit training (HICT, n=6) groups. Statistical analysis showed negative correlations between CAT and vigorous PA (VPA) (*r*_s_=-0.41, *p*=0.037); and between percent body fat (%BF), fat mass (FM), and all PA levels (*r*_s_=-0.41– -0.68, *p*<0.05); while muscle mass was positively associated with moderate-to-vigorous PA, and upper-body lean mass with all PA levels (*r*_s_ =0.40–0.53, *p*<0.05). The HICT intervention showed significant improvements (*p*<0.05) in %BF, FM, fat free mass, and whole-body and lower extremities lean mass and strength after three weeks; however, only leg strength and upper extremities’ FM improved significantly compared to CON and HICT. In conclusion, although all types of PA showed a positive influence on body fat content, only VPA significantly impacted on CAT volume. Moreover, three weeks of HICT induced positive changes in PFit in women with obesity. Further research is needed to explore VPA levels and high-intensity exercise interventions for short- and long-term CAT management.

## 1 Introduction

Obesity is a known risk factor for cardiovascular disease (CVD), diabetes, hypertension, and other diseases and it is characterized by excess adipose tissue (1–3). In normal conditions, Cardiac Adipose Tissue (CAT) accumulates in two distinct depots adjacent to the myocardium and within the pericardium: epicardial adipose tissue (EAT) (4) and pericardial adipose tissue (PAT) (5). EAT is mostly deposited at perivascular interventricular and atrioventricular grooves and has the same embryological genesis as intra-abdominal visceral adipose tissue (VAT) (6, 7), while PAT is deposited between the two pericardial layers (visceral and parietal) as well as on the parietal pericardium’s exterior surface (8).

Under normal physiological conditions, CAT deposits protect the heart by secreting various molecules (e.g., anti-inflammatory and anti-atherogenic cytokines), however, excess CAT has been postulated as a distinct pathologic feature for CAD development (9) due to its altered biochemical activity (10–12). Abnormal CAT accumulation stimulates the release of inflammatory cytokines [i.e. interleukin (IL)-1, IL-6, IL-8, IL-11, TNF-α (tumor necrosis factor-α), IL-16, IL-17, G-CSF (granulocyte colony stimulating factor and granulocyte-macrophage colony-stimulating factor (GM-CSF)] that could lead to the progression of atherosclerosis in coronary arteries, myocardial infarction, heart failure, atrial fibrillation, arrythmias, and other adverse cardiac events (13–16). As a consequence, excess CAT surrounding the myocardium has gained attention as a risk factor for CVD and other medical complications (e.g., metabolic syndrome, visceral adiposity, heart morphology, insulin resistance, sub-clinical atherosclerosis, or liver enzymes) (13, 17, 18). More specifically, some research has linked PAT to coronary high-risk lesions (19). In this context, quantification and prevention of CAT accumulation has become an important target for disease prevention and treatment (20).

Various methods exist to estimate body fat (e.g., anthropometric measures, dual x-ray, magnetic resonance imaging, and radiation-exposing computed tomography) (21–23). Cardiac magnetic resonance (CMR) imaging is considered a gold standard technique for measuring CAT. However, the selective quantification methods (such as single slice vs. multiple slice sampling, or regional vs. total) impact accuracy of adiposity evaluation (24, 25). Therefore, total CAT volume should be accurately measured – rather than estimated – by analyzing the entire thoracic region around the heart.

Currently, the relationship between physical activity (PA) levels, physical health, and CAT accumulation is unknown (26). There is, however, a substantial correlation between sedentary behaviors and the risk of metabolic disease and CVD (27) as well as both cardiovascular-specific mortality and total mortality (28). This association is largely caused by vascular factors (i.e. vascular dysfunction, downregulating blood flow, altering glucose metabolism, and activating inflammatory and oxidative stress pathways) (29, 30). Likewise, observational research in PA levels has shown a negative relationship with fat mass (FM) and dispersion (31, 32). The use of practical, accurate, and reliable tools for evaluating PA can help to identify the relationship between PA and disease and assist in the study of effect modification (32–34).

In this context, vigorous exercise has shown to improve anti-inflammatory and metabolic function, and cardiovascular health on a dose response fashion (35–37). A recent meta-analysis on randomized controlled trials comparing short- to medium-term (2-16 weeks) supervised exercise programs concluded that exercise can reduce CAT ‘considerably’ in overweight individuals or those with obesity (38); however, the duration of interventions was not associated to the magnitude of the effect on CAT. Lastly, the effect of exercise mode was not evaluated given the scarcity of resistance-based intervention studies. In physically inactive people with abdominal obesity, both aerobic and resistance training (RT) reduced CAT when meeting the recommendations for intensity and frequency (39–44). However, RT seems to be the only mode of exercise capable of reducing PAT (43, 44). Interestingly, these studies did not report nor matched aerobic and RT interventions by energy expenditure, hence, not allowing for realistic comparisons (45, 46).

The goal of this study was to analyze the relationships between PA, PFit, and CAT volume in obese women, and to pilot test the differential effects when comparing energy expenditure-matched high-intensity interval aerobic and resistance training interventions.

## 2 Materials and methods

**Figure 1:**
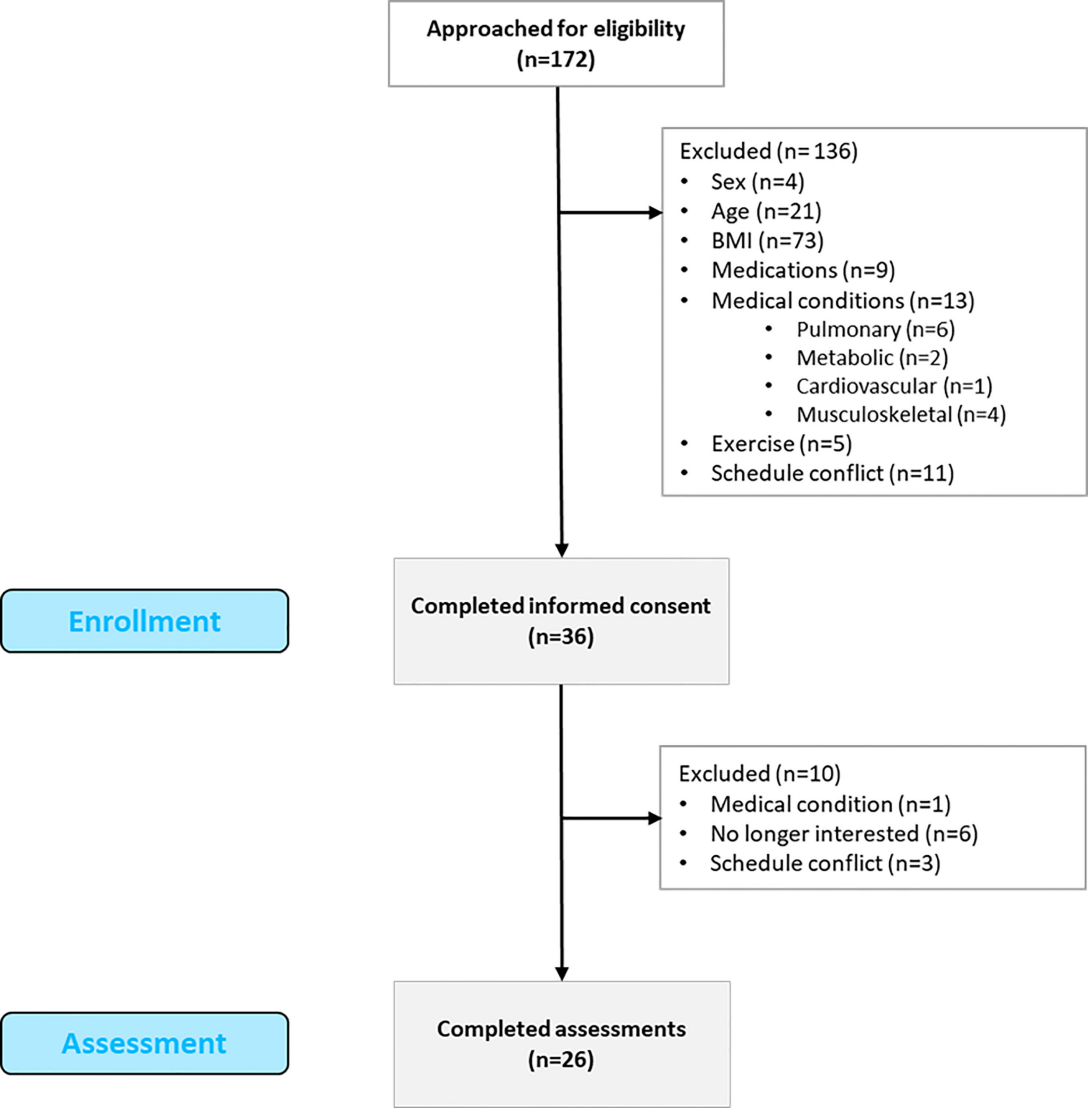
Flow diagram.

This study followed an observational cross-sectional design and was approved by the Institutional Review Board (ID: # 16-1208-4C). The reporting of observational studies (STROBE) recommendations was followed (47) The study was conducted between 2017 and 2020 in accordance with the Declaration of Helsinki ethical principles. A total of 172 individuals were contacted to participate in the study (see **Figure 1**). Inclusion criteria were: 18 to 45 years of age, female, BMI from 30 to 39.99 kg/m^2^, and sedentary lifestyle. Exclusion criteria were as follows: males, cardiovascular, metabolic, pulmonary, or musculoskeletal conditions, pregnant women, smokers, those taking medications, and individuals with schedule incompatibilities. A total of 136 participants were excluded for not meeting the criteria: 4 were male, 21 did not meet the age range, 73 participants did not meet the BMI criteria, 9 were on medications, 13 presented medical conditions, 11 were unable to participate due to a schedule conflict, and 5 participated in regular exercise. Thirty-six participants signed the consent form and were enrolled in the study. A total of 10 participants were discontinued due to loss of interest, schedule conflicts, and previously non-reported medical conditions. Finally, a total of 26 participants completed the cross-sectional study.

**Figure 2:**
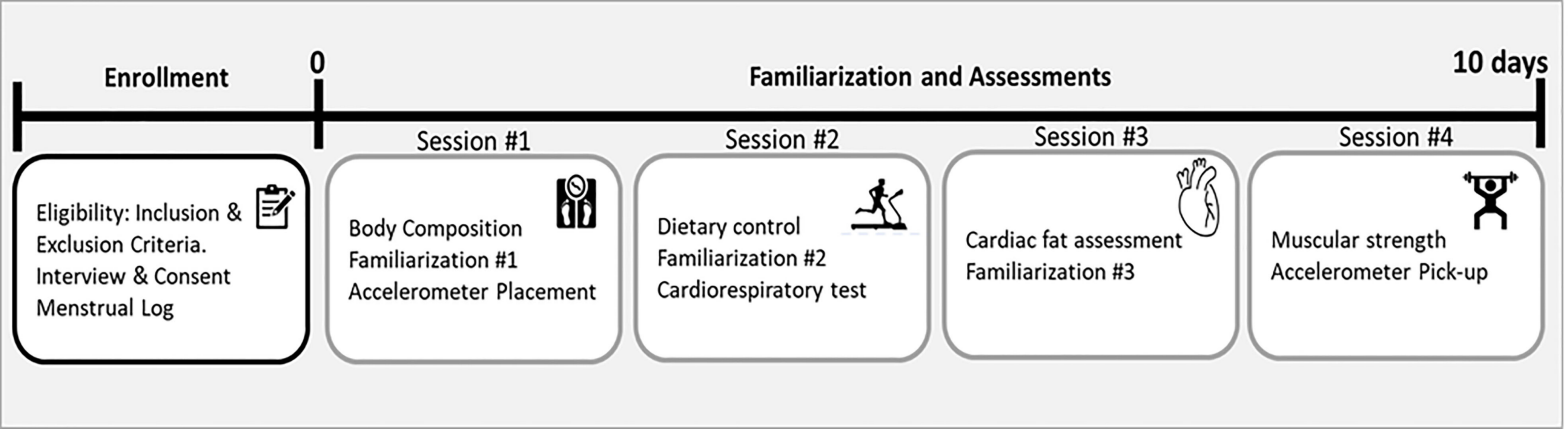
Study design.

Assessments were performed in four sessions with a minimum of one day of rest between them (see **Figure 2**). All assessments were performed coinciding with the transition between two menstrual cycles. To determine the menstrual cycle, each participant was required to fill in a menstrual log documenting the length of three previous menstrual cycles (48).

### 2.1 Physical activity assessment

Participants were asked to wear an Actigraph GT3X accelerometer for seven days. The device was worn on the right hip and held in place *via* an elastic belt. Participants were instructed to wear continuously while awake and to remove it only when sleeping, bathing, or swimming. A minimum of three days was required to be valid. General guidelines were followed as for minimum wear time (49–51). Relative sedentary time (%ST) was reported, and activity was classified as either sedentary, light, moderate, vigorous, or very vigorous as defined by Freedson (52).

### 2.2 Physical fitness assessment

*Muscular strength test*. To reduce the learning effect, ensure proper technique for each exercise, prevent injuries, and teach participants how to use the OMNI-Resistance Exercise Scale (OMNI-RES from 0 to 10), subjects completed three familiarization sessions during the first week of the project (53). Each familiarization session included a warm-up and cool-down period, as well as a conditioning phase consisting of 2–3 sets of 5–8 repetitions of non-weighted exercises. The one maximum repetition (1RM) test was performed following standardized procedures following the familiarization sessions (54, 55).

*Cardiorespiratory testing*. Cardiorespiratory fitness was measured breath by breath (Parvo Medics’ TrueOne 2400, Sandy, UT, USA) on a treadmill (Trackmaster TMX425C, Newton, KS, USA) using the conventional Bruce methodology (56) up to 85% of the maximal heart rate (HR).

*Body composition and anthropometric assessment.* Fat mass content (FM), percent body fat (BF), fat free mass (FFM), and lean body mass (LBM) were measured using dual-energy X-ray absorptiometry (GE Lunar Dual Energy X-ray Absorptiometry iDXA, Madison, WI, USA) in accordance with manufacturer instructions. Skeletal muscle mass (SMM) was calculated (57). The waist and hip circumferences, as well as the body weight (BW), were all measured. BMI was calculated as weight/height^2^ (kg/m^2^), waist-to-hip ratio (WtH) as waist/hip, and waist-to-height ratio (WtHR) as waist/height. At the beginning of the study and after three weeks of training, the same researcher measured each participant.

### 2.3 Dietary controls

*Dietary controls*: Dietary habits were assessed using the Automated Self-administered 24-hour recall (ASA24h) at the beginning of session two (see **Figure 2**). Total energy intake, proteins, total fats, saturated fatty acids (SFA), monounsaturated fatty acids (MUFA), polyunsaturated fatty acids (PUFA), carbohydrates, and sugars were reported (58).

### 2.4 Cardiac adipose tissue assessment

*Magnetic Resonance:* A 3.0T MRI system was used to acquire cardiac MRI data (Vida, Siemens Healthineers, Erlangen, Germany). Electrocardiogram (ECG) and respiratory-navigator-gated MRI scans were performed in 1-cm increments along the cardiac short axis over the entire heart. The sequence used had an in-plane resolution of approximately 1.47 mm^2^. The volumes were created by obtaining still-frames corresponding to the diastole from the cine loops. The volumes were loaded into 3DSlicer, an open-source medical imaging software, and the CAT was manually identified on each slice from the apex to the mitral valve. The CAT volume in mm^3^ was computed by multiplying the number of CAT voxels identified by the size of each voxel (~1.47mm x 1.47 mm x 10mm).

*Echocardiography*: A standard set of parasternal and apical echocardiographic images were obtained with the volunteer lying in the left lateral decubitus position. The echo-free space between the external wall of the myocardium and the visceral pericardium was identified as epicardial fat. The parasternal long-axis view images were utilized for quantification, but parasternal short-axis images were used when quality of the long-axis view was suboptimal. The thickness was measured on the surface of the right ventricle perpendicular to the free wall at end systole along the midline of the ultrasound beam, perpendicular to the aortic annulus in a total of three cardiac cycles (59).

### 2.5 Pilot intervention study

The pilot intervention was registered at www.clinicaltrials.gov (ID: NCT03297333), and interventions were carried out following baseline evaluations (starting at early follicular phase). The objective was two-fold, first, to examine the differential effects of high-intensity interval training (HIIT) and high-intensity circuit training (HICT), and second to calculate the appropriate sample size for a larger scale randomized control trial. A total of 18 participants joining the pilot intervention study and were blocked by age, BMI, and %BF, and randomized to either HIIT, HICT, or non-exercising control (CON) group (n= 6 each group) (60). Two participants were lost: one refused to continue when allocated to the CON group and one participant in the HIIT group started taking contraceptives. The intervention was suspended due to the COVID-19 (Corona virus) pandemic.

*Supervised exercise programs* were conducted three times per week for three weeks. Exercise sessions included a 10-minute warm-up and cool-down including dynamic and static stretching exercises of major muscle groups, respectively. Based on prior findings a goal was to reach an energy expenditure of ~500 kcals/session (43). The core of the HICT sessions consisted of a circuit of seven exercises performed in the following order: LP (leg press), barbell bent-over row, back squat, weighted crunches, deadlift, BP (bench press), and weighted squat jumps. Further details on this training protocol are described elsewhere (43). The intensity was set to a minimum of 7 of the OMNI-RES to ensure an intensity of 70–75% of 1RM after each exercise (53), and the circuit was repeated until the target energy expenditure was achieved. The HIIT sessions consisted of five-minute intervals divided into two periods where the intensity was controlled based on each participants’ HR and the Borg’s rating of perceived exertion (RPE). The first period of the interval included three minutes of high-intensity activity at 70-85% of predicted HR_max_ and an RPE between 13-17 (15-17 at the end of the period), and the second period included two minutes of moderate intensity (60-65% of HR_max_) and an RPE between 10-12 (61). Both HR and energy expenditure were monitored using a Polar FT4 (Polar Electro, Inc., NY, USA). The number of circuits and intervals completed was registered throughout all sessions. Participants in the CON group did not take part in the exercise sessions, and all groups were requested to maintain their physical activity and eating habits throughout the trial.

### 2.6 Statistical analyses

Data are shown as mean and standard deviation (SD), unless otherwise stated. A Shapiro-Wilk test was performed to verify the normal distribution of the variables, where normality was assumed if p > 0.05. Associations between CAT volume and levels of PA and PFit were examined by Pearson’s and Spearman correlation analyses, depending on normal or non-normal distribution. Non-parametric Mann-Whitney U test and related samples Wilcoxon signed-rank test were utilized to analyze between groups and within group effects of the pilot intervention, respectively. The strength of the associations (rs) and effect sizes (r) were classified as ≤ 0.1 (very small/very weak), 0.1- 0.29 (small/weak), 0.3- 0.49 (moderate), 0.5- 0.69 (high/strong), 0.7- 0.89 (very high/very strong), and 0.9- 1 (perfect). The level of significance was set at *p* < 0.05. The Statistical Package for the Social Sciences (SPSS) version 20.0 (SPSS Inc., Chicago, IL, USA) was used to perform all the statistical analyses.

## 3 Results

### 3.1 Association study

The mean age of the participants was 23.5 ±6.0 years of age. Data on PFit, PA, and diet are reported as means, standard deviations, minimum and maximum values as shown in **Table 1**.

**Table 1:** Characteristics of the sample (n=26).

	Mean		SD	Min	Max
*Anthropometry*					
** Height (m)**	1.6	±	0.1	1.5	1.8
** BW (kg)**	89.4	±	10.8	69.5	107.5
** BMI (kg/m^2^)**	33.6	±	2.6	30	39.4
** Waist (cm)**	104.4	±	15.9	84.1	147.5
** Hip (cm)**	121.2	±	13.2	109	163.3
*DXA*					
** % BF**	47.8	±	3.6	40.9	55.1
** FM (kg)**	40.8	±	6.6	31.9	57.2
** LBM (kg)**	44.5	±	5.8	31.5	53
** BMC (kg)**	2.7	±	0.5	1.2	3.6
** FFM (kg)**	46.8	±	5.9	33.6	56
** TA-Fat ratio**	0.9	±	0.1	0.7	1
** VAT (cm3)**	450.5	±	545.7	0.4	1830
** SMM (kg)**	28.2	±	4	20.1	34.8
Cardiac-MR					
** CAT (mL)**	68.1	±	32.4	25.8	139.8
*Muscle Strength*					
** 1RM-LP (kg)**	127.4	±	31.4	79.5	205
** 1RM-BP (kg)**	43.9	±	12.3	29.5	95
*Functional Capacity*					
** rVO_2_est (mL/kg/min)**	21.8	±	4.1	10.2	28.4
** aVO_2_est (l/min)**	2	±	0.3	1.5	2.9
*Physical Activity*					
** LPA (min/day)**	358.8	±	335.6	31.8	1076.5
** MPA (min/day)**	162.5	±	144.8	15.4	531.5
** VPA (min/day)**	3.4	±	6.4	0	31.2
** MVPA (min/day)**	130.7	±	147.5	3.6	541.6
** %ST**	91.3	±	4.4	83.8	98.8
*Diet*					
** Energy (kcal/day)**	1647.0	±	648.1	438.9	3145.4
Proteins					
** Protein %**	20.9	±	6.1	12.0	34.1
** Protein (g)**	70.0	±	34.9	9.7	174.4
Fats					
** Total Fat %**	21.9	±	7.2	8.4	40.5
** Total Fat (g)**	74.0	±	43.0	6.6	180.1
** SFA %**	6.9	±	2.9	2.2	13.8
** SFA (g)**	23.2	±	11.4	5.0	57.6
** MUFA %**	7.6	±	2.7	2.7	13.9
** MUFA (g)**	25.6	±	12.9	7.6	61.9
** PUFA %**	5.6	±	2.5	2.0	10.9
** PUFA (g)**	19.0	±	10.4	3.6	47.6
CHO					
** CHO %**	57.2	±	11.3	32.4	79.6
** CHO (g)**	192.1	±	89.7	27.8	460.4
** Sugars %**	21.3	±	10.6	3.8	46.2
** Sugars (g)**	76.3	±	54.3	2.6	224.0

CON, control group; HIIT, high-intensity interval training; HICT, high-intensity circuit training; BW, Body weight; BMI, Body Mass Index; %BF, % body fat; FM, Fat mass; LBM, Lean body mass; BMC, Bone Mineral Content; FFM, Free Fat Mass; TA-Fat ratio, (Appendicular + Trunk) to total Fat ratio; VAT, Visceral Adipose Tissue; SMM, Total Body Skeletal Muscle; CAT, Cardiac Adipose Tissue; 1RM-LP, 1 Max Rep Leg Press; 1RM-BP, 1 Rep Max Bench Press; rVO2, Relative VO2 peak; aVO2, Absolute VO2; LPA, Low Physical Activity; MPA, Moderate Physical Activity; VPA, Vigorous Physical Activity; MVPA, Moderate to Vigorous Physical Activity; %ST, percent sedentary time; SFA, Saturated Fatty Acids; MUFA, Monounsaturated Fatty Acids; PUFA, Polyunsaturated Fatty Acids; CHO, Carbohydrates.

Association analyses between CAT, PA, PFit levels, and dietary macronutrients and sugars revealed moderate to high (0.3 – 0.7) significant associations (*r*_s_). An association matrix table is available as **Supplementary Table S1**. CAT and VPA levels (*r*_s_ = - 0.41, *p* = 0.037) were negatively associated, while positive associations were found between CAT and waist circumference (*r*_s_ = 0.58, *p* = 0.002) and VAT (*r*_s_ = 0.60, *p* = 0.001). No other associations between CAT and muscular or cardiorespiratory fitness were found.

Analyses examining the PA data showed positive associations between all levels of activity and upper-body lean mass (LPA: *r*_s_ = 0.52, *p* = 0.007; MPA: *r*_s_ = 0.53, *p* = 0.005; VPA: *r*_s_ = 0.40, *p* = 0.044; MVPA: *r*_s_ = 0.65, *p <*0.000). Similarly, MVPA levels positively impacted lower-body lean mass (*r*_s_ = 0.45, *p* = 0.021) and SMM (*r*_s_ = 0.43, *p* = 0.029). In addition, PA levels were negatively associated with %BF (LPA: *r*_s_ = - 0.65, *p* < 0.001; MPA: *r*_s_ = - 0.62, *p* = 0.001; VPA: *r*_s_ = - 0.41, *p* = 0.037; MVPA: *r*_s_ = -0.68, *p <*0.000) and lower-body FM (LPA: *r*_s_ = - 0.49, *p* = 0.011; MPA: *r*_s_ = - 0.42, *p* = 0.034; VPA: *r*_s_ = - 0.43, *p* = 0.027). Lastly, only LPA showed negative correlations with the heart rate reached at end-test (*r*_s_ = - 0.44, *p* = 0.024), and %ST was positively associated with lower-body FM (*r*_s_ = - 0.41, *p* = 0.039).

Diet composition analyses revealed positive associations between daily energy intake and waist circumference (*r*_s_ = 0.51, *p* = 0.008), VAT (*r*_s_ = 0.42, *p* = 0.034), upper-body muscle function (*r*_s_ = 0.45, *p* = 0.019) and relative oxygen consumption (*r*_s_ = 0.40, *p* = 0.043). In addition, carbohydrate and sugars intake correlated negatively with MPA (*r*_s_ = - 0.40, *p* = 0.043, and *r*_s_ = - 0.42, *p* = 0.034, respectively), and LPA was negatively associated with carbohydrate intake (*r*_s_ = - 0.40, *p* = 0.042).

### 3.2 Pilot study

Participants in the pilot study had a mean age of 23.7 ±7.6 years, a BMI of 32.7 ±1.7 kg/m^2^, a %BF of 44.1 ±2.1%. No significant differences were found for age, BMI, and %BF between groups (CON, HIIT and HICT) at baseline. Moreover, diet-related variables were similar between groups at the beginning of the pilot study (**Supplementary Table S2**). When comparing duration of exercise sessions (HIIT: 51.1 ±5.1 min; HICT: 53.9 ±6.0 min) and derived energy expenditure (HIIT: 534.7 ±15.6 kcals/session; HICT: 530.1 ±21.2 kcals/session) between exercising groups no significant differences were found (*U* = 9, z = - 0.640, *p* = 0.522, *r* = 0.19; and *U* = 8, z = - 0.853, *p* = 0.394, *r* = 0.26, respectively).

The nonparametric Wilcoxon test for related samples revealed significant changes in body composition and muscular health (muscle mass and function) with very strong effect sizes (0.83 – 0.90) only for the HICT group. Specifically, %BF and FM were significantly reduced after three weeks of intervention (Z = - 2.207, *p* = 0.027, *r* = 0.90; Z = - 2.201, *p* = 0.028, *r* = 0.90, respectively). Furthermore, LBM (Z = - 2.201, *p* = 0.028, *r* = 0.90), FFM (Z = - 2.201, *p* = 0.028, *r* = 0.90), and lower extremities lean mass and strength (Z = -2.023, *p* = 0.043, *r* = 0.83; Z = - 2.023, *p* = 0.028, *r* = 0.90, respectively) improved significantly. No differences were found for CON, while the HIIT group showed a significant increase in WtH ratio (Z = - 2.023, *p* = 0.043, *r* = 0.90).

The Mann-Whitney U test revealed a significant increase of WtHR in the HIIT group compared to controls (*U* = 7.0; z = - 1.149; *p* = 0.009; *r* = 0.37). Percent change in leg strength (1RM-LP) was significantly superior in the HICT group compared to controls (*U* = 0; z = - 2.739; *p* = 0.006; *r* = 0.00) and FM in the upper extremities was significantly reduced in HICT compared to HIIT (*U* = 8.0; z = - 1.095; *p* = 0.049; *r* = 0.33). Effect sizes varied from weak to strong (0.02 – 0.58). No other significant differences were found. **Table 2** shows mean values at baseline and percent change of body composition, muscular health, and cardiorespiratory fitness.

**Table 2:** Effects of the pilot interventions on body composition, muscular health, and cardiorespiratory fitness.

	CON (n=5)						HIIT (n=5)							HICT (n=6)					
	baseline			% change			Baseline			% change			baseline				% change		
	Mean		SD	Mean		SD	Mean		SD	Mean		SD	Mean			SD	Mean		SD
Anthropometry																			
** BW (kg)**	87.2	±	7.7	0.5	±	2.1	84.8	±	8.9	0.2	±	1.3	88.3		±	7.0	-1.3	±	1.6
** BMI (kg/m^2^)**	32.8	±	2.2	0.6	±	1.8	32.5	±	1.4	0.0	±	1.5	32.8		±	1.8	-1.6	±	1.7
** Waist (cm)**	97.8	±	12.6	-0.4	±	1.4	94.7	±	12.7	0.9	±	7.6	99.8		±	9.6	-3.7	±	4.0
** Hip (cm)**	113	±	5.0	0.8	±	2.1	109	±	13.2	-4.2	±	9.1	113.7		±	4.9	-1.9	±	4.0
** WtH ratio**	0.9	±	0.1	-1.2	±	0.9	0.9	±	0.1	5.6	±	3.8	0.9		±	0.1	-1.8	±	4.6
** WtHR ratio**	0.6	±	0.1	-0.4	±	1.3	0.6	±	0.1	0.8	±	7.5**^a^	0.6		±	0.1	-3.7	±	4.1
DXA																			
** % BF**	43.8	±	2.8	-1.2	±	3.7	43.4	±	1.8	-1.4	±	1.2	45.1		±	1.5	-3.7	±	2.2
** FM_total_ (kg)**	36.6	±	4.1	-0.9	±	5.2	35.3	±	3.8	-2	±	3.5	38.1		±	3.3	-4.7	±	3.1
** FM_Legs_ (kg)**	12.4	±	1.4	-3.4	±	4.9	13.0	±	2.5	-0.5	±	1.8	12.2		±	1.6	-3.3	±	2.2
** FM_Arms_ (kg)**	4.2	±	0.7	-4.2	±	8.1	3.5	±	0.6	2	±	5.4	4.5		±	0.6	-4.1	±	7.5*^b^
** LBM (kg)**	47.1	±	5.3	1.3	±	2	46.1	±	5.5	-0.8	±	3.0	46.6		±	3.1	1.8	±	1.8
** LM_Legs_ (kg)**	17.5	±	1.8	3.1	±	3.5	16.8	±	2.6	0	±	1.8	16.9		±	1.3	2.7	±	2.1
** LM_Arms_ (kg)**	5.4	±	0.9	-2.3	±	6.4	4.9	±	0.8	0.9	±	3.8	5.4		±	0.8	3.1	±	7.8
** FFM (kg)**	48.2	±	6.4	1.8	±	2.3	47.9	±	5.1	2.5	±	5.4	47.8		±	3.7	1.7	±	1.9
** SMM (kg)**	30.0	±	3.3	0.7	±	2.6	28.8	±	4.5	-0.6	±	2.1	29.5		±	2.6	1.3	±	1.3
Muscle Strength																			
** 1RM-LP (kg)**	106	±	32	-1.2	±	11	128	±	45	14.4	±	15	105		±	17	28.9	±	16**^c^
** 1RM-BP (kg)**	43.1	±	11	6.2	±	7	52.1	±	24	-1	±	6	40.8		±	6.5	13.1	±	11
Functional Capacity																			
** rVO_2_est**	29.8	±	8.1	0.5	±	29	27.2	±	4.2	7	±	7.9	29.4		±	5.3	4.7	±	21
** aVO_2_est**	2.6	±	0.8	0.4	±	28	2.3	±	0.2	4.4	±	8.1	2.6		±	0.5	4.6	±	22

CON, control group; HIIT, high-intensity interval training; HICT, high-intensity circuit training; BW, Body weight; BMI, Body Mass Index; WtH, Waist to Hip; %BF, % body fat; FM, Fat mass; LBM, Lean body mass; LM, Lean Mass; FFM, Free Fat Mass; SMM, Total Body Skeletal Muscle; 1RM-LP, 1 Max Rep Leg Press; 1RM-BP, 1 Rep Max Bench Press; rVO_2_est, Estimated relative maximal oxygen consumption in ml/kg/min; aVO_2_est, Estimated absolute maximal oxygen consumption in L/min.

*p < 0.05; ** p < 0.01.

a CON versus HIIT.

b HIIT versus HICT.

c CON versus HICT.

Data reported as means and standard deviations.

**Figure 3:**
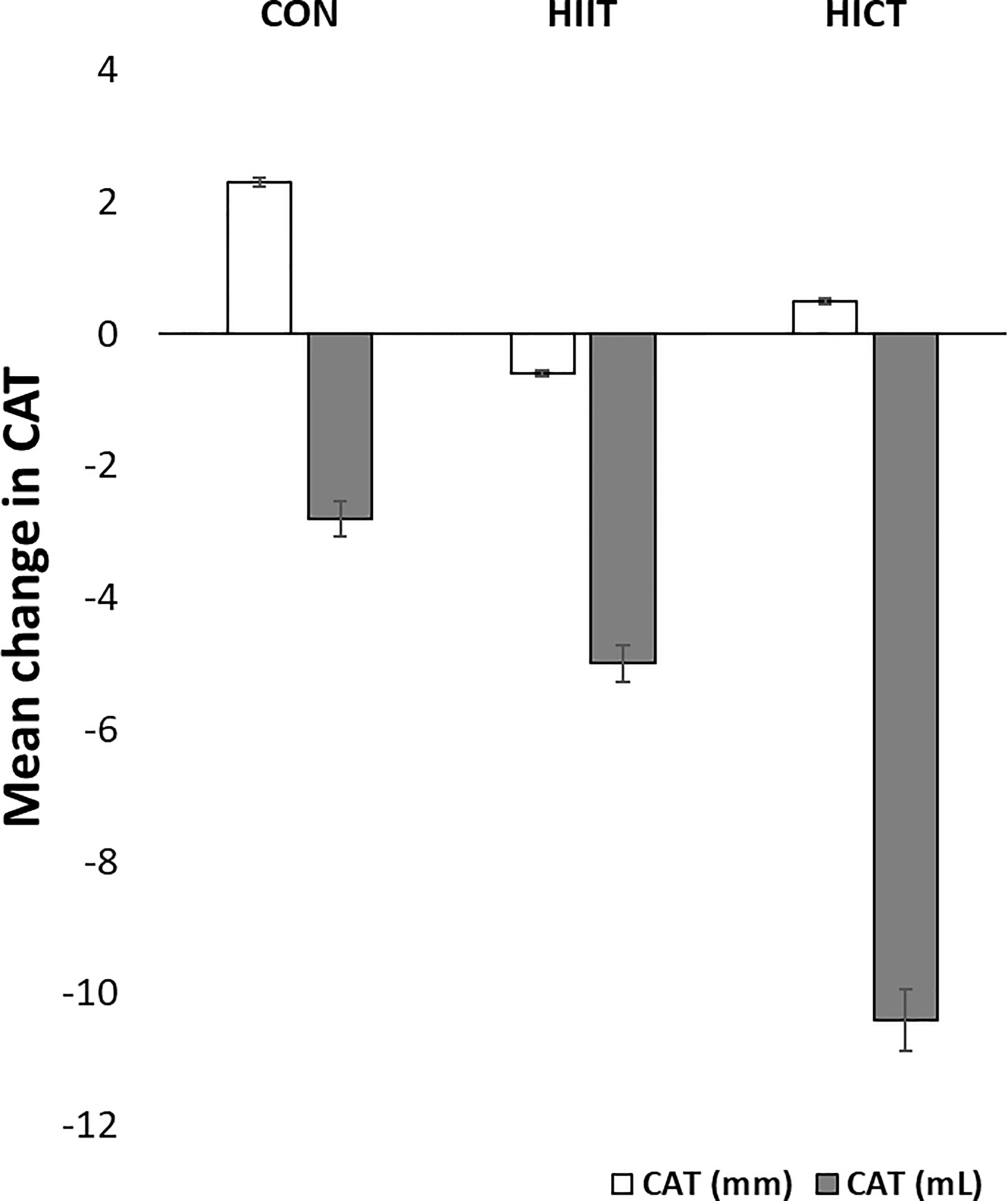
Change in CAT. CON – control group; HIIT – high intensity interval training group; HICT – high intensity circuit training group; CAT (mm) – cardiac adipose tissue derived from echocardiography analysis; CAT (mL) – cardiac adipose tissue derived from magnetic resonance analysis. Data reported as means and standard error of mean.

**Figure 4:**
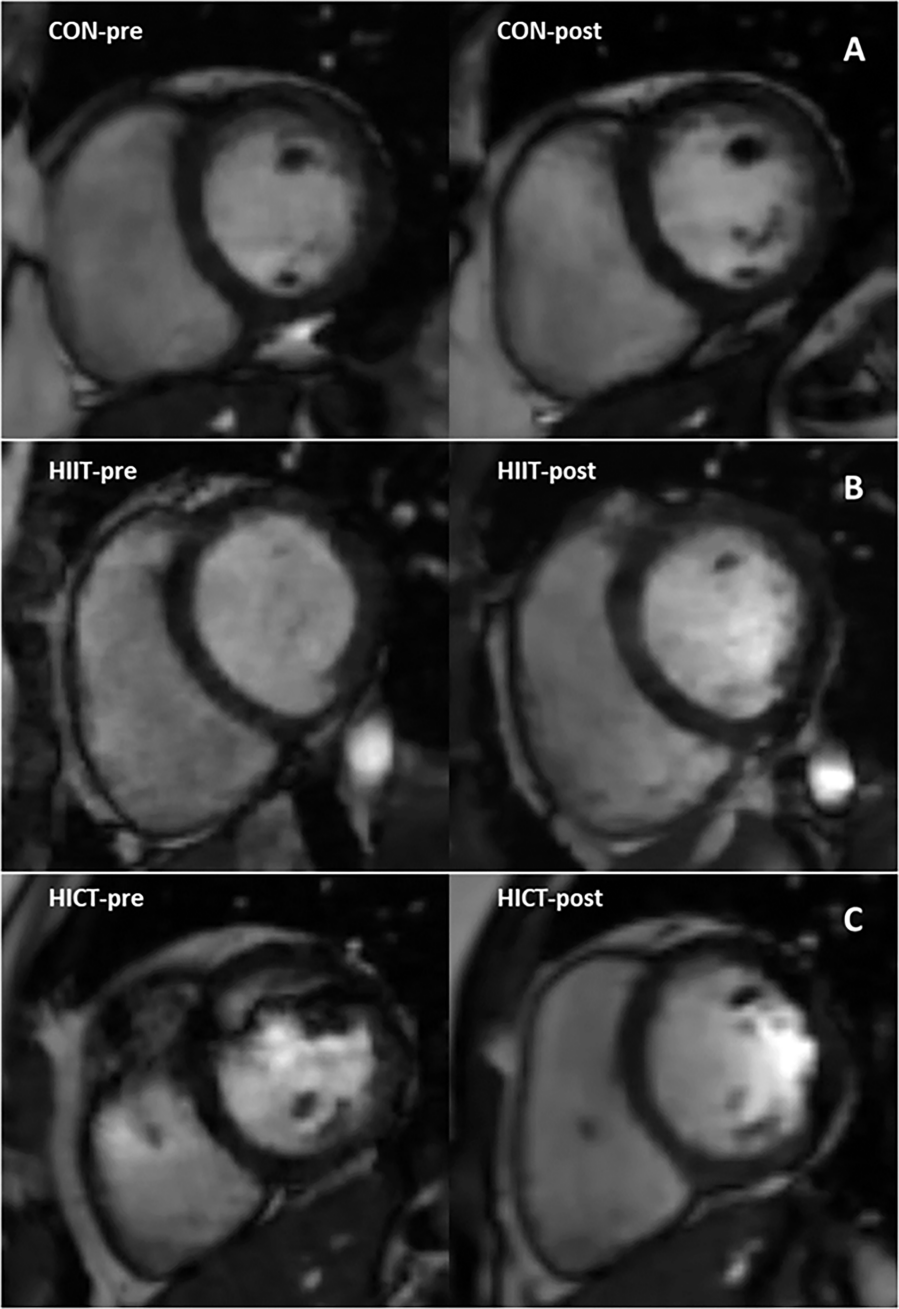
Magnetic resonance images representing pre- to post-intervention changes in cardiac fat of three participants. Close-up of the cardiac fat for a single subject in: **(A)** the control group; **(B)** the HICT group; and **(C)** the HIIT group. HICT – high-intensity circuit training; HIIT – high-intensity interval training.

Baseline MR-derived CAT values (CON: 58.5 ± 1.2 mL; HIIT: 39.9 ± 1.6 mL; HICT: 68.7 ± 0.9 mL) and echocardiography-derived CAT values (CON: 5.9 ± 0.09 mm; HIIT: 4.2 ± 0.16 mm; HICT: 5.1 ± 0.04 mm) were similar at baseline. The Mann-Whitney U test did not show significant differences between groups on change in CAT thickness (echocardiography) or volume (MR). **Figures 3**, **4** show, respectively, the change in MR-derived and echocardiography-derived CAT and three MR-CAT scans representative of control, HIIT and HICT groups. Individual participant changes in CAT from all the groups (CON, HIIT and HICT) derived from both MRI and echocardiography are presented as **Supplementary Figure S1**.

## 4 Discussion

Overall, this study revealed that all types of PA are negatively associated with relative whole-body and lower-body fatness. Furthermore, this is the first study to reveal a significant negative link between CAT and VPA in women with obesity. In this study, only HICT showed significantly positive effects in body fat and lean mass content (i.e. %BF, WC, FM, LBM, FFM, lower-extremity LBM) and muscular strength after just three weeks of training. Since this is an exploratory study, larger samples are required to detect differences between groups (HIIT *versus* HICT) and determine which exercise modality has the greatest impact on CAT. When considered as a whole, our data highlight VPA and high-intensity exercise modalities as potential key modulators for the management of CAT and total adiposity. As CAT deposits are becoming identified as risk factors for CVD development, preventative measures to minimize CAT accumulation are crucial (62). Therefore, our findings are clinically relevant as both VPA and HICT could be valuable strategies for health optimization.

Studies in activity-related behaviors have revealed strong relationships between reduced PA, increased sedentary time and obesity (63, 64). For example, activity time and MVPA are associated with lower levels of adiposity and BMI, and a decreased risk for obesity (65, 66). Excessive ST >75% (>9 hours/day) has been associated with poor body composition profile and lower muscle mass (67, 68). Conversely, light and higher intensity PA is favorably related with increased lean mass (69). Our results are in agreement with these authors as %ST was associated with increased levels of lower-body fat, while all PA levels were linked to greater upper-body lean mass and reduced %BF and lower-extremities fat content.

To the best of our knowledge, no other studies have reported nor analyzed the associations between CAT and VPA levels. Analysis of the body composition revealed positive associations of CAT with WC and VAT that is consistent with the literature (70–72). For example, Sironi et al. reported fat accumulation and increased CAT to be proportional to the degree of obesity (72). The *Framingham Heart Study* also revealed that high WC and VAT was more frequent in individuals with increased CAT (70, 71). Despite these observations, CAT was only associated with vascular calcification (70). Furthermore, insulin resistance (73), and serum free fatty acid levels (74) have been connected to increased EAT and CAT, respectively. The existing data supports the idea that whole CAT (EAT and PAT) may have harmful perivascular consequences (70). Research has shown that higher EAT in those with coronary artery disease triggers inflammation and could play a role in the formation of plaque by secreting cytokines that are pro-inflammatory. Consequently, EAT is a biomarker of cardiovascular risk (75). Additionally, the volume of pericardial fat is highly correlated with plaque eccentricity, supporting that pericardial fat is associated with atherosclerosis (76).

Regarding diet’s composition, participants diet was not different from current guidelines for Americans, except for sugars 21.3% which were above the 10% recommended (77). As for the relationship with PA, low levels of PA have been postulated as a risk factor for poor dietary habits and fat gain. Indeed, Shook and collaborators revealed that those individuals with the lowest PA levels consumed higher amounts of calories and had ~2–4 higher-risk of “gaining clinically significant amounts*”* of fat over one year (78). Supporting these results, our data showed a positive association between daily calorie intake and both WC and VAT. Interestingly, our study also revealed positive correlations between both upper-LBM and all levels of PA. These results in agreement with previous research, as low PA levels have been consistently associated with poor functional capacity and muscle performance in normal weight, overweight and obesity (79).

Our data revealed negative correlations between consumption of both sugar and carbohydrates and MPA and LPA levels. Other studies have linked low levels of PA and increased sedentary behaviors (e.g., increased TV, video and computer time) with high sugar intake and other unhealthy dietary habits (e.g., poor vegetable and fruit consumption) (80, 81). Although we did not find significant relationships between sugar intake and CAT, prior research analyzing various ectopic fat deposits – “excess adipose tissue in locations not classically associated with adipose tissue storage” (82) such as VAT, PAT or EAT – revealed positive associations between sugar intake, and PAT and VAT volumes (83). The study by Yi and collaborators included 3070 participants studied over a 20-year period. Moreover, research has exposed that the diets rich in sugar my trigger feelings of exhaustion and loss of energy, attributing to the detrimental impact on PA and sedentary behaviors (84). However, the underlying mechanisms that sugars seem to have on PA and sedentary habits or *vice versa* are still unknown.

There is considerable interest in learning how to prevent CAT accumulation. Recent studies in the effectiveness of exercise interventions, have shown CAT reduction associated with improvements in FM or %BF (43, 85) lipid profile (86), ST (87), and inflammatory markers (85, 88) in response to exercise. More importantly, resistance and aerobic exercise interventions have shown to reduce EAT in those individuals with abdominal obesity, but only high-intensity resistance exercise has been capable of decreasing PAT (43, 44). Overall, exercise intensity prescribed was insufficient and did not meet the recommendations for weight loss (225-420 min/week) (89), and the change in CAT after 12-24 weeks of exercise was <10% (~1 mm) (90–93) except for the high-intensity interventions (43, 94). In addition, only three studies utilized resistance-based exercise for 12 weeks (44, 95) and 3 weeks (43), two utilized a high-intensity circuit format (43, 44), and only supervised interventions resulted in large reductions in CAT volume (43, 44). Similar to current findings, our research group reported a decrease in CAT (>10%) and PFit improvement (i.e. %BF and muscle function) after a three weeks of a high-intensity progressive resistance training (RT). The intervention was conducted in young adult females with obesity (BMI=34.13 ± 3.16 kg/m^2^) and included three RT sessions per week targeting large muscle groups (43). Another program targeting primarily small muscle groups (e.g., arm curls, leg extensions, leg curls, triceps extensions, seated row and leg press) allowed for a rather modest CAT (~1 mm, thickness) reduction (95). Thus, resistance-based exercise programs of higher intensity appear to be key for CAT loss. In a randomized control trial, Jo and collaborators examined the impact of eight weeks, three times per week, of HIIT and moderate-intensity continuous training (MICT) on endothelial function and CAT content in individuals with hypertensive metabolic syndrome (94). The HIIT group exercised for 3 minutes at 40% heart-rate reserve (HRR), followed by 3 minutes at 80% HRR; whereas in the MICT group, participants exercised for 3 minutes at 60% of HRR. All sessions lasted 30-40 minutes. Although, patients in both groups significantly reduced CAT (HICT, *p* = 0.001; MICT *p* = 0.01), the effect of HIIT was larger. However, these differences cannot be attributed solely to intensity, as the duration of the programs and mode of exercise differed. In our study, energy expenditure and intensity were matched, and duration of sessions was similar. Therefore, we could speculate that the differences could have been caused by the exercise mode (HIIT vs HICT) implemented. Supporting our findings, Christensen and collaborators uncovered that only resistance exercise induced reductions in both EAT and PAT compared to HIIT that only reduced EAT (44). The mechanisms underlying these selective changes are unknown; however, it is known that ectopic fat deposits have functional and structural characteristics (96) (e.g., adrenergic receptor-mediated lipolysis, etc.) that enable significant responses to higher-intensity exercise. Nevertheless, this hypothesis has to be investigated.

Our results emphasize the importance of the selection of an adequate exercise “dose” (i.e. frequency, intensity and time), mode (i.e. resistance, endurance), and format (i.e. traditional vs circuit format). Circuit training is a particularly well-liked exercise program style due to its ability to combine high-intensity resistance and aerobic interventions (97). Contrary to the WtHR increases found in the HIIT, researchers comparing high-intensity versus low-intensity interval exercise have reported improvements in body composition with higher intensities (97). Based on prior research, this unusual finding could have been HIIT-mediated by increased feelings of hunger (98). However, this remains a question as no dietary-records were collected during or after the intervention period. Supporting our results, resistance circuit-based training for an average duration of 10 weeks (range: 4–28) significantly reduces FM while increasing muscle mass, and upper and lower body strength (99). Furthermore, HICT has been successful in improving (+6.3%) cardiorespiratory fitness (maximal oxygen consumption or VO_2_max), (+0.3%) maximum aerobic speed or power, and (+2.6%) aerobic performance (99). Accordingly, our pilot HICT intervention showed a trend toward superior gains in lower body muscle strength and larger reduction in WtHR compared to HIIT.

This research is not without limitations. One important limitation is the sample size which limits the generalization of our results, so caution should be used when interpreting both CAT volume associations and the optimal exercise mode (HIIT versus HICT) to reduce CAT. The reason behind this was the interruption caused by COVID-19 (Corona virus) pandemic. Furthermore, in order to avoid the probability of committing type II error (given the small sample size) we did not perform Bonferroni correction; however, this might have increased the risk of a type I error linked to multiple comparisons. This exploratory research did not include *a priori* sample size calculations which are essential to avoid underpowered or overpowered studies. Nevertheless, the sample was enough to reveal novel information related to the potential association between CAT and VPA. Likewise, the use of a 24-hour recalls has associated limitations (e.g., it is limited to the foods listed, only registers the last 24 hours, requires the participant to remember all food consumed, etc.) that could have caused the loss of information concerning dietary habits. Future research in VPA and CAT should include larger samples, so regression model analysis is viable and moderate to high effect sizes are achieved in association and interventional studies. Then, results could be generalized to the population.

Some important features strengthen this study. The research was designed following the criteria for cross-sectional and randomized control trials. The sample was homogeneous (women with obesity but otherwise healthy) and menstrual cycle was monitored to ensure all participants were evaluated during the same hormonal phase. In addition, samples on the pilot intervention (CON, HICT and HIIT) were equivalent at baseline. Another feature that strengthens this exploratory study is that the intensity prescribed in this program met the recommendations, and energy expenditure was monitored and matched in both HIIT and HICT; hence, allowing for realistic comparisons. Lastly, total CAT was measured *via* cardiac MRI assessment rather than estimated which provides a more precise quantification; likewise, results obtained with DXA showed cardiac fat changes were accompanied by changes in body composition.

## 5 Conclusions

All types of PA have a positive influence on whole-body and lower-body fat and muscle content. However, only VPA shows potential to significantly impact CAT volume. Moreover, short-term high-intensity exercise strategy in women with obesity shows promise, as three weeks of HICT induced positive changes in their body composition and muscular health. Future research is needed to explore vigorous intensity activity and high-intensity interval exercise interventions as tools for physical fitness optimization, CAT reduction and CVD risk management in women with obesity short- and long-term.

## Data availability statement

The raw data supporting the conclusions of this article will be made available by the authors, without undue reservation.

## Ethics statement

The studies involving human participants were reviewed and approved by Institutional Review Board (ID: # 16-1208-4C) at Southern Illinois University Edwardsville. The patients/participants provided their written informed consent to participate in this study.

## Author contributions

MF-d-V and JK conceived and designed the research, and PW, BS, PD, and AG edited and revised the manuscript. BSS, PD, BS, AG, PW, JK and MF-d-V performed the experiments. MF-d-V, JP-R, JK and ST analyzed the data. MF-d-V, ST and JP-R interpreted the results of the experiments, MF-d-V, ST and JK prepared figures, and MF-d-V, ST, JP-R, BS and JK drafted the manuscript. MF-d-V, BSS, PD, BS, AG, JP-R, PW, and JK approved the final version of the manuscript.

## Supplementary material

Supplementary Figure 1 Individual changes of CAT by group. CON, control group; HIIT, high intensity interval training group; HICT, high intensity circuit training group; CAT (mm), cardiac adipose tissue derived from echocardiography analysis; CAT (mL), cardiac adipose tissue derived from magnetic resonance analysis.
