# Supplementary figures and images for: Vigorous-intensity exercise as a modulator of cardiac adipose tissue in women with obesity: a cross-sectional and randomized pilot study
Source: Front Endocrinol (Lausanne). 2023 May 8;14:1104441. doi: 10.3389/fendo.2023.1104441 (PMC10200876; doi:10.3389/fendo.2023.1104441)

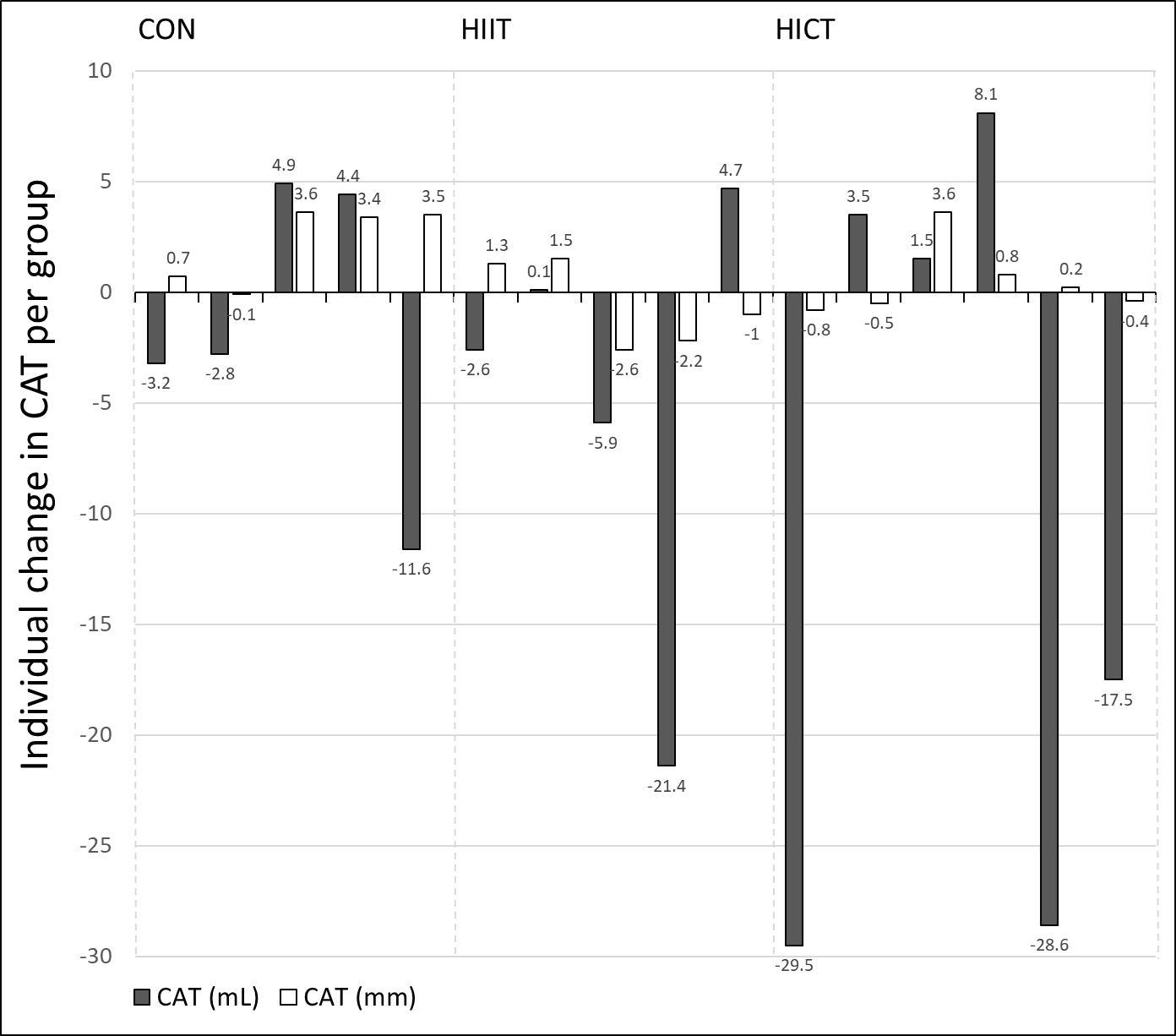

Supplement: Supplementary Figure 1 — Individual changes of CAT by group. CON, control group; HIIT, high intensity interval training group; HICT, high intensity circuit training group; CAT (mm), cardiac adipose tissue derived from echocardiography analysis; CAT (mL), cardiac adipose tissue derived from magnetic resonance analysis. [file Image_1.tif]
